# Dr. Evans's Case of Fracture of the Cranium

**Published:** 1800-10

**Authors:** J. Evans

**Affiliations:** Ketley, Shropshire


### *To the Editors of the Medical and Physical Journal.*

GENTLEMEN,

SEVERAL instances of Fractures of the Skull doing well without the use of the trephine, having been published by Mr. O'Halloran, Mr. Abernethy, and other men of character in their profession, I trust every additional fact on the subject may prove useful to the young and inexperienced practitioner; therefore, I take the liberty of communicating the following Case that lately came under my care, which, if you think worthy of a place in your valuable Journal, is much at your service. I am,

GENTLEMEN,

Your obedient humble servant,

J. EVANS.

*Ketley, Shropshire,*

*Sept.* 15, 1800.

---

On the 21st of December last, Thomas Haywood, a boy, 9 years of age, being at work in a coal mine in this neighbourhood, a large quantity of coal fell upon his shoulders when he was in a stooping posture, by which he was driven with considerable force upon his head, against an iron rail that formed one side of a way for the conveyance of small carriages from the remote parts of the pit to the bottom of the shaft. In consequence of this accident, an extensive portion of the scalp was detached from the occipital bone; and about an inch above the orbit of the right eye, there was a large wound of the integuments, accompanied with a fracture of the frontal bone, *three* inches in *length*, with a depression of the lower portion of it, *three-eights* of an inch below its original level.* The superior edge of the fracture was very rough, a loose fragment of which I removed with my thumb and finger. The boy vomited,

* The depth of the depression was measured on the tenth day after the accident, at the time the plasters were removed, when it appeared much less than at first.

mited, and talked a little incoherently while I was dreffing him, but foon recovered, fo as to give rational anfwers to fuch queftions as were put to him. As he had no alarming fymptom of compreffed brain, I did not think myfelf juftifiable in making ufe of the trephine; therefore, brought the retracted edges of the wound together with futures, and applied flips of adhefive plafter over the whole, with an intention of retaining the parts more firmly in contact. The detached portion of fcalp on the pofterior part of the head (after being freed from a confiderable quantity of coal flack, which had been forced under it) was replaced, and fecured in its fituation with fticking plafter, and a bandage lightly applied. After his wounds were dreffed, he was well enough to be conveyed home, a diftance of half a mile from my houfe, where he had been brought immediately after the accident. A dofe of Dover's powder was given him that night, which had a good effect; but the next morning he complained of much pain in the injured parts. The fcalp being tumefied, I directed the bandage to be conftantly moiftened with a folution of crude fal ammoniac in vinegar; by this mode of treatment the pain and fwelling foon abated.

The plafters were not removed from the forehead for the fpace of ten days, at which time they became loofe in confequence of a fmall difcharge of matter; the wound appeared then in a healthy granulating ftate; that on the occiput healed principally by the firft intention. It is worthy of remark, that the boy was not confined to his bed a fingle day, nor did one unfavourable fymptom occur. Opiates and cathartics were occafionally adminiftered; and in the fpace of five weeks he was perfectly well without any exfoliation, notwithftanding a confiderable part of the os frontis was denuded both above and below the fracture.

